# Experimental and Therapeutic Opportunities for Stem Cells in Multiple Sclerosis

**DOI:** 10.3390/ijms131114470

**Published:** 2012-11-08

**Authors:** Rickie Patani, Siddharthan Chandran

**Affiliations:** 1 Anne McLaren Laboratory for Regenerative Medicine, Stem Cell Institute, University of Cambridge, Forvie Site, Robinson Way, Cambridge CB2 0SZ, UK; E-Mail: rp379@cam.ac.uk; 2 Cambridge Centre for Brain Repair, Department of Clinical Neurosciences, University of Cambridge, Forvie Site, Robinson Way, Cambridge CB2 0PY, UK; 3 Euan MacDonald Centre, University of Edinburgh, Chancellor’s Building, 49 Little France Crescent, Edinburgh EH16 4SB, UK

**Keywords:** multiple sclerosis (MS), embryonic stem cells (ESCs), pluripotent stem cells (PSCs), induced pluripotent stem cells (iPSCs), neurodegenerative disease

## Abstract

Multiple Sclerosis (MS) is an inflammatory demyelinating neurodegenerative disorder of the brain and spinal cord that causes significant disability in young adults. Although the precise aetiopathogenesis of MS remains unresolved, its pathological hallmarks include inflammation, demyelination, axonal injury (acute and chronic), astrogliosis and variable remyelination. Despite major recent advances in therapeutics for the early stage of the disease there are currently no disease modifying treatments for the progressive stage of disease, whose pathological substrate is axonal degeneration. This represents the great and unmet clinical need in MS. Against this background, human stem cells offer promise both to improve understanding of disease mechanism(s) through *in-vitro* modeling as well as potentially direct use to supplement and promote remyelination, an endogenous reparative process where entire myelin sheaths are restored to demyelinated axons. Conceptually, stem cells can act directly to myelinate axons or indirectly through different mechanisms to promote endogenous repair; importantly these two mechanisms of action are not mutually exclusive. We propose that discovery of novel methods to invoke or enhance remyelination in MS may be the most effective therapeutic strategy to limit axonal damage and instigate restoration of structure and function in this debilitating condition. Human stem cell derived neurons and glia, including patient specific cells derived through reprogramming, provide an unprecedented experimental system to model MS “in a dish” as well as enable high-throughput drug discovery. Finally, we speculate upon the potential role for stem cell based therapies in MS.

## 1. Introduction

Multiple Sclerosis (MS) is a chronic inflammatory and progressive demyelinating disorder of the central nervous system that causes significant morbidity. It has a prevalence of 1.2 per 1000 and a lifetime risk of 1 in 400 [1]. Although an interplay of genetic and environmental factors are likely to be contributory, the precise aetiopathogenesis of MS remains unresolved. Its pathological hallmarks include multi-focal inflammation, primary demyelination (where axons pathologically lose their investing myelin sheaths), acute and chronic axonal damage and astrogliosis. The majority of patients experience two disease phases; relapsing-remitting (RR) followed by a secondary progressive (SP) course. The former is pathologically characterised by inflammatory activity while SPMS is dominated by neurodegeneration and variable remyelination. Major recent advances in anti-inflammatory disease modifying treatments (DMTs) have transformed the outlook of newly diagnosed RRMS patients. However, whether these DMTs will influence the later progressive stage that accounts for accumulating disability is unknown. In addition approximately 15% of patients experience a progressive course from diagnosis that again is not responsive to currently available DMTs. The unmet need is therefore for novel neuroprotective therapeutics.

## 2. Repair in MS

MS is the prototypic immune-mediated demyelinating disease characterized pathologically by mult-focal and multi-phasic inflammatory demyelinating lesions of the central neuraxis. Blood-brain barrier breakdown and subsequent (multi-) focal inflammation typically leads to clinical disability in the form of discrete relapses. In addition to targeting a particular neural cell type (oligodendrocytes-responsible for myelination in the central neuraxis), MS additionally exhibits regional bias with particular sites of predilection, including the optic nerves, periventricular white matter, cerebellum and corpus callosum. Most MS patients develop progressive and permanent neurological deficit after 10–15 years of disease activity. Axonal loss and progressive brain atrophy are histologically and radiologically conspicuous features of MS [2–6], and comprise the pathological substrate for accumulating neurological disability. Studies exploring axonal pathology/loss have reported its occurrence in both acute and chronic MS plaques and even in normal appearing white matter [2–4,7]. Precise underlying mechanisms remain unresolved but these aforementioned studies and others argue for both inflammation-related [8] and inflammation-independent mechanisms of axonal loss.

The major therapeutic goal in MS is neuroprotection; two important components of this goal are the promotion of remyelination [9] and the prevention of axonal loss [10]. *In vivo* studies demonstrate that these two processes are intricately coupled, and suggest that remyelination will serve to protect axons [11–13] in addition to restoring saltatory conduction. To this end, the human central nervous system does possess some capacity for variable degrees of remyelination, which can even be extensive in some cases [14,15]. Although remyelination results in thinner and shorter myelin sheath “internodes” than would be expected for a given diameter of axon [16,17], its potential as a reparative strategy is clearly demonstrated by experimental association with resolution of function deficits in animal models [18,19]. Unfortunately, however, remyelination ultimately fails to keep pace with disease progression and neurological deficit accumulates. Understanding why endogenous remeylination appears to fail in some patients and is variable across different lesions in the same individual is critical to guiding therapeutic strategy.

Following an episode of demyelination, sodium channels (usually concentrated at the nodes of Ranvier) redistribute as a compensatory mechanism thus allowing conduction to be maintained [20,21] (and conduction block avoided). The resulting action potentials are, however, delayed and continuous rather than fast and saltatory. In addition to ensheathing/myelinating axons, oligodendrocytes also contribute to axonal stability, axonal length and neurofilament regulation, sodium channel clustering and neuronal survival [22–30]. The observation that specific genetic defects affecting myelin lead to axonal degeneration in mice [31,32] reinforces the notion that axonal survival ultimately requires oligodendrocyte-mediated trophic support. Indeed in MS post-mortem tissue, axon preservation is seen in remyelinated lesions; again reinforcing the concept of a supportive role for myelin in axon protection [12]. Such studies raise the hypothesis that there are oligodendroglia-derived factors that protect axons; indeed insulin like growth factor 1 (IgF1) and glial derived neurotrophic factor (GDNF) have been shown to be produced by oligodendrocytes in cell culture where they do exert axon-protective effects [29,30]. The mechanistic advantages for promoting remyelination are thus not only to restore saltatory conduction; but also to protect the axon from “bystander” inflammatory damage (a concept supported by considerable albeit indirect experimental evidence) [12,32,33].

Cell-based remyelination is the focus of many experimental studies. This can theoretically be accomplished by exogenous promotion of endogenous remyelination or more directly by exogenous myelinogenic cells, although it is important to appreciate that these two mechanisms are not mutually exclusive. Despite classic dogma from early neuroanatomists like Cajal, the adult mammalian CNS does indeed contain populations of resident, proliferating and multipotent neural stem cells [34]. These adult stem cells are concentrated in the subventricular zone and hippocampus, and are also diffusely distributed throughout the neuraxis in the form oligodendrocyte precursor cells (OPCs) [35,36]. The OPC may well have the potential to behave as a neural stem cell in the context of injury, representing a new approach to brain repair. An increasing body of evidence suggests that remyelinating oligodendrocytes arise from adult OPCs [37–42]. Whether this represents a completely homogenous precursor population within the adult neuraxis has yet to be resolved; region specific molecular and functional diversity within the OPC population is certainly possible and will greatly inform future efforts to generate such cells for modeling disease mechanisms and for their potential use as a therapeutic strategy.

**Figure 1:**
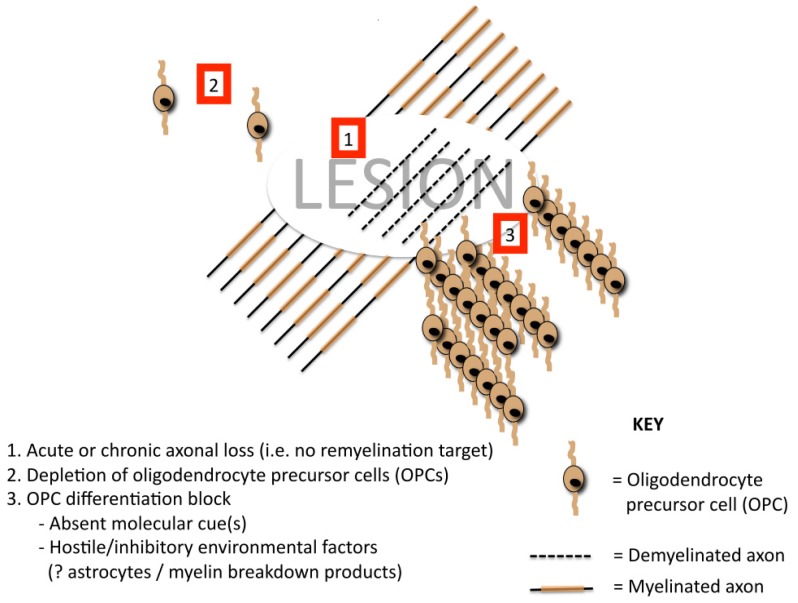
Putative mechanisms of remyelination failure.

Why remyelination fails in some lesions but not others is unknown [43]. Some of the putative mechanisms for remyelination failure are depicted in Figure 1. Clearly the preservation of axonal integrity within a lesion is a key determinant of successful remyelination (*i.e.*, there must be viable “targets” to remyelinate). The repopulation of demyelinated areas by adult OPCs is robust [44], suggesting that repeated bouts of demyelination/remyelination to individual foci should not subsequently deplete OPC reserve and cause remyelination failure [45]. It has been argued, however, that if there is insufficient “recovery” time between episodes of inflammatory demyelination (*i.e.*, repeated / continuous disease activity), this may well contribute to remyelination failure [46,47]. A failure of OPC recruitment has also been raised as a possible mechanism of remyelination failure [48] and is supported by the finding of patients who generate antibodies to OPC-expressed antigens [49] or aberrant expression of local guidance cues [50]. Differentiation block of intralesional premyelinating oligodendrocytes has also been implicated as a contributor to remyelination failure [51–54]. The presence of myelin debris containing myelin associated differentiation inhibitory proteins also reduces the efficiency of remyelination [55]; age related decline in the macrophage response may be relevant here [56,57]. Changes in molecular expression on demyelinating axons have again been implicated in remyelination failure [58,59], as has intralesional astroglial phenotype [60–62]. Additional factors influencing the efficiency of experimental remyelination include age [57], sex [63] and genetic background [64].

Pathological studies from patients with early and aggressive multiple sclerosis have proven to be a valuable resource for understanding early pathological events [65,66], including those associated with remyelination failure. Neuropathological studies of such (hyper-) acute lesions exhibiting remyelination failure have been associated with the following phenomena: presence of differentiated oligodendrocytes, astrocyte/oligodendrocyte interaction (emperipolesis), malformed (nodular) myelin sheaths, active demyelination of remyelinated lesions (“second hit” lesions). Importantly, the pattern of active demyelination appears to be the same in all newly forming lesions. Remyelination failure appears to begin early in the course of MS, and occurs following the reappearance of differentiated oligodendrocytes in recently demyelinated tissue (Patani, Barnett and Prineas, unpublished observations). These findings reinforce the importance of early intervention to overcome remyelination failure, but also highlight the need to understand the mechanisms underpinning this phenomenon [48].

Detailed pathological analyses of more chronic progressive MS cases have also yielded valuable insights including an association of progressive disease with insoluble tau accumulation [67,68], not seen in early aggressive disease [66]. Further similar studies are necessary not only to refine current lesion taxonomy, but also to deepen our understanding of the cellular and molecular temporal evolution of demyelinating lesions. Additionally, it is critical to better understand the precise mechanisms of endogenous repair and how these might be optimally invoked during the early phase of disease. Indeed, the molecular basis of remyelination is now beginning to be elucidated; by using stage-specific comprehensive transcriptional profiling of spontaneous remyelination in a rodent model, a recent study found that retinoid acid receptor RXR-γ transcripts were differentially expressed in cells of the oligodendrocyte lineage. The study, using a variety of methods, went on to show that RXR-γ is a positive regulator of endogenous oligodendrocyte precursor cell differentiation and remyelination [69], raising the prospect of retinoid pathway manipulation as a pharmacological target for regenerative therapy in MS.

## 3. Experimental Models to Understand Disease Mechanisms and Evaluate Therapeutic Approaches

Classical and indispensible experimental systems employing *in vivo* (transgenic or lesion-based models; Table 1) and cell line studies are unable to comprehensively recapitulate the complexity and precise biology of the human system. Despite considerable evolutionary conservation between vertebrates there are important differences that need to be addressed in order to improve the translational yield of experimental studies. In addition to accumulating evidence for developmental, anatomical and functional evolutionary divergence between rodents and primates [70–73], intricate cellular processes including neuroprotective pathways have also been shown to differ [74,75], and these findings further reinforce the importance of complementary human experimental approaches [76]. An important major interspecies difference is the proportion of glia within the nervous system, which is far higher in humans; a fact that is of particular interest in the current review.

Additionally, therapeutic strategies initially shown to be efficacious in animal disease models have proved unsuccessful in several cases when translated to pre-clinical and clinical human trials [77–80]. Therefore, human systems are required to complement existing experimental models in discovering disease mechanisms and therapeutic targets of direct relevance to human disease. Such model systems are accessible to some extent through human diagnostic tissue biopsy samples and post-mortem specimens. However, limited tissue availability, cellular viability, effects of post-mortem delay, and crucially the difficulty of obtaining material that accurately recapitulates early stages of the disease process [81], are major limitations of this strategy.

Against this background, the isolation of human embryonic stem cells (hESCs) [82] and subsequent directed differentiation to regionally defined neurons [83–89] and glia [90–95] coupled with recent developments in nuclear reprogramming [96–98] have generated unprecedented experimental opportunities around using human stem cells to both better understand mechanisms underlying neurological diseases such as MS, and for use in their treatment. More specifically, it is now possible to create reductionist *in vitro* myelination co-culture systems to precisely elucidate mechanisms of demyelination, axonal injury and remyelination. Application of various injury paradigms (e.g., nutritional deprivation, oxidate stress) to these co-culture models can be utilized to gain further insight into the molecular pathogenesis of MS as previously described [28–30,99]. Such human *in vitro* myelination co-culture systems will facilitate the discovery of novel therapeutics in a more focused and clinically relevant manner. Such approaches will complement existing and indispensible *in-vivo* model systems.

## 4. Stem Cells

A stem cell is defined as having the ability to both self-renew and generate specialised cell types. The differentiation of a stem cell to a specialized cell type is often simplified to a series of phenotypically defined populations, but in fact this is a continuum with multiple specific genetic steps (which may overlap). Human stem cells are of value as both an experimental resource (to study disease mechanisms and for drug discovery) and, potentially, a therapeutic strategy. Their ability to generate almost unlimited numbers of potentially clinical grade region-specific functional neuronal and glial populations while remaining karyotypically stable makes them a valuable resource.

A common taxonomy of stem cells is based on the developmental stage of isolation, which in turn determines the repertoire of specialised cell types they can generate (or their “potency”). Stem cells isolated from early stages of development possess greater potency than those from later in development. Embryonic stem cells (ESCs) are a type of pluripotent stem cell (PSC) that posses the greatest potency *in vitro,* responding predictably to developmental cues. Human ESCs (hESCs) were first isolated by Jamie Thompson’s laboratory in 1998 [82] from the inner cell mass of the blastocyst from where cells were subsequently grown in culture using established methods [100–102]. The introduction of chemically defined hESC culture and differentiation has strengthened the prospect of establishing clinical-grade cells for use in regenerative medicine [103,104].

Initial attempts to derive pluripotent stem cells by different methods were largely inspired by early nuclear transfer experiments from Sirs John Gurdon and Ian Wilmut [105,106]. These efforts focussed on nuclear reprogramming by somatic cell nuclear transfer (SCNT) and cell fusion techniques [107]. A more recent method of generating patient-specific PSCs is based on the discovery that somatic cell nuclei e.g., Fibroblasts can be directly reprogrammed to an embryonic stem cell-like pluripotent state without the need for eggs through introduction of a quartet of transcription factors. These can be introduced into somatic fibroblasts using standard methodology available in many labs [98]. These cells are referred to as induced pluripotent stem cells (iPSCs). Importantly, this approach has also been demonstrated using human somatic cells [97]. Induction of the pluripotent state originally necessitated transduction (viral transfection) of four transcription factors, including the oncogenic *c-myc* transcription factor. However, progress has since been made to improve efficiency and/or reduce the need for genetic manipulations [108–110]. Human iPSCs thus offer a unique opportunity to derive patient specific cell lines to model neurological diseases such as MS. These recent and transformative developments in reprogramming biology have demonstrated the practical feasibility of deriving patient specific functional cell types from readily accessible patient somatic cells, either directly by forward programming, trans-differentiation or via an induced pluripotent state [97,98,111,112].

Human ESCs and iPSCs thus represent a powerful and unparalleled experimental opportunity to model neurodegenerative disease by virtue of their competence to respond to developmental signals permitting specification of functional neurons and glia. Thus far, directed differentiation has been achieved to various region-specific fates including human spinal cord, midbrain and forebrain neurons [83–85,87,113,114]. These studies support the faithful recapitulation of spatio-temporally regulated developmental responsiveness to appropriate extrinsic morphogenetic signals permitting systematic manipulation of cell fate. Although this represents a significant advance, the next challenge is understanding how highly refined sub-region specific neuronal and glial diversity is generated. Recent studies suggest that *in vitro* human stem cell based systems can begin to capture this diversity [89,90]. Establishing how faithfully such differentiated cell populations resemble their somatic counterparts (foetal and adult) is also critical in understanding their true utility to accurately model disease [115].

Human iPSCs offer an advantage over hESCs in enabling study of patient specific lines including disease-causing mutations from routine manipulation of readily available patient material [81,116]. The promise of stem cells as tools for understanding the mechanisms of neurological disease has been realized more so than their promise for neurological repair. To date, proof-of-concept studies using iPSCs have recapitulated the pathological phenotype of neurons from patients with inherited and sporadic developmental and adult brain disorders that include spinal muscular atrophy [117], familial dyautonomia [118], Rett syndrome [119], Parkinson’s disease [120] and Motor neuron disease [121]; and have provided novel insights into disease mechanisms and potential therapeutic targets.

Compared to ESCs, stem cells isolated at later developmental stages (e.g., foetal stem cells) possess restricted phenotypic potentials that are defined by both the tissue and the tissue sub-region from which they are isolated. Several studies have demonstrated that these cells cannot be readily directed towards a myelinating cell fate, a desirable attribute in demyelinating conditions such as MS [122–125]. Given the numbers of cells required for experimental or therapeutic application, regional restriction represents a potential problem because this cannot, at present, be predictably manipulated using extrinsic signals.

## 5. Human Pluripotent Stem Cells (hPSCs) as a Disease Model System for MS

The concept of cell autonomous *vs.* non-cell autonomous mechanisms of disease is of considerable importance in this context, and can be directly addressed using a hPSC-based model system. One major challenge of using such an approach for the study of MS disease mechanisms is the difficulty of reliably generating oligodendrocytes from hPSCs [73], although this has been achieved—using different methods—by several groups [92,93,95]. Indeed, studies have already highlighted evolutionarily divergent signaling pathways in deriving oligodendrocytes from human compared to mouse ESCs, from which oligodendrogenesis and subsequent expansion of precursor populations is more easily achieved [95]. Given the likely interplay between genetic factors and environmental influences in MS, deriving iPSCs from this patient group may well be of value. Reductionist experimental paradigms may include generating oligodendrocytes from iPSCs derived from an MS patient and modulating the *in vitro* environment to model injury and repair in MS. Oligodendrocytes can then be used to myelinate neurons derived from the same hPSCs. Such myelination co-cultures can then be perturbed using injurious stimuli known to be relevant in MS in order to elucidate the precise mechanisms disrupting integrity of the neuron–oligodendrocyte interaction. Systematically and incrementally increasing the complexity of such co-culture systems will ultimately allow precise recapitulation and understanding of the key pathological phenomena in MS.

Similar studies using rodent systems demonstrate the practical feasibility and conceptual value of such approaches; they have elegantly identified some of the mechanisms by which oligodendrocytes are neuroprotective [29,30], including in the context of nitric oxide mediated neuronal and axonal injury [28]. Others have utilized different co-culture paradigms using activated CNS microglia and neurons and explored the mechanisms of injury and neuroprotection in this context [99]. “Genome editing” of the disease-specific iPSC line by the use of zinc finger nuclease mediated tailored genome engineering could be used to establish the influence of gene candidates identified from previous genetic studies [8,31,32] on iPSC-derived oligodendrocyte vulnerability [126–130]. Such approaches have significant potential to unravel the complexities of temporal dynamics and aberrant molecular events in different phases of MS lesion evolution. Furthermore, they raise the prospect for drug discovery by high throughput *in vitro* screening.

The dynamic and evolving role of astrocytes in neurodegeneration and neuroprotection is another particularly relevant example of cell autonomous *vs.* non-cell autonomous mechanisms of disease. The recent paradigm shift in motor neuron disease being (at least in part) an astrogliopathy [131–133] highlights the importance of cellular autonomy studies. It is also possible to explore the role of cell autonomous *vs.* non-cell autonomous mechanisms of oligodendrocyte injury and protection using similar strategies in co-culture paradigms (e.g., myelinated neurons with mutant *vs.* healthy astrocytes). The emerging neuroprotective role of astrocytes is also both of great interest and potential relevance neurodegenerative disease [134], although this has yet to be systematically explored in the context of MS. Further sophistication of experimental design could be to explore the vulnerability of region-specific oligodendrocytes or isotopic (similar/same regional identities) *vs*. anisotopic (different regional identities) co-culture paradigms. There is some evidence from using isotopic (midbrain) foetal astrocytes in co-culture with ESC derived midbrain dopaminergic neurons, which suggests that such experiments will be informative [135].

## 6. Stem Cell Therapy in MS

Broadly, stem cell therapy could work by two mechanisms, which are not mutually exclusive: (i) exogenous cell replacement and (ii) by enhancing endogenous repair.

### 6.1. Exogenous Cellular Repair

Once the desired cell type (OPC) has generated it must be rigorously characterized to qualify as a viable therapeutic candidate. The cells must have the robustness to survive and migrate through the pathological host milieu in order to myelinate denuded axons, exist is sufficient numbers, retain to capacity to proliferate and avoid immune rejection, while not causing tumor formation. Successful remyelination has been achieved experimentally in a range of animal models and using a variety of cell types. These have included embryonic- and adult-derived cells of the oligodendrocyte lineage, Schwann cells, olfactory ensheathing cells (OECs), and neural precursors (NPCs) and non-NPCs [41,136–144]. OECs and Schwann cells are the only potentially autologous adult human cell populations with myelinating potential. OECs promote and augment axon growth [145] and may also have a role in myelin repair [136,139,146,147]. Olfactory ensheathing cells appear to be able to migrate through and more readily integrate into an astrocytic environment [148,149], which is clearly advantageous in MS given the pathological hallmark of astroglial scar formation. Schwann cell possess the theoretical advantage of being antigenically distinct, thus resistant to ongoing injury in MS, to oligodendrocytes.

However, the translatability of these findings in acute monophasic rodent models to human MS, which is a relapsing-remitting chronic inflammatory condition, is less clear. Recognition of different lesion pathological phenotypes [150] also raises the question of whether different bespoke reparative strategies may be necessary. Furthermore, different disease courses (and indeed distinct phases within one disease course) may necessitate different therapeutic considerations [151]. Additionally, there is evidence that immune cells have diverse roles in the context of lesion evolution; there is evidence that macrophage depletion inhibits remyelination in experimental models [56] and in human post mortem studies, macrophage density at lesion borders has been positively correlated with remyelination [15]. These and other insights from experimental studies and post-mortem human pathological analyses indicate that more acute lesions represent optimal targets for transplantation-mediated repair, given the relative preservation of axons as targets for remyelination [15,55,56,152,153]. They also bring into question current clinical approaches to managing relapses (do high doses of steroids—which will effectively “deplete” macrophages in addition to other arms of the immune system—have a deleterious effect on endogenous reparative mechanisms?). Clearly the temporal dynamics of depletion and repopulation together with the optimal timing of remyelination and interplay between macrophages and other cell types, are important considerations to comprehensively address this particular question.

Multiple specific genetic steps contribute to the transition from stem cell to a terminally differentiated state, raising the question of which specific point in the differentiation process provides cells best suited to the desired therapeutic goal. Successful therapeutic strategies require consideration of both (i) the pathogenic environment into which cells are to be deployed, and (ii) the desired therapeutic action (e.g., immunomodulation, stimulation of endogenous repair systems, or cell replacement). For example, an inflammatory environment has been shown to promote astrocyte specification from NPCs [154], whereas transplantation of OPCs into this environment results in functional remyelination [153]. Assuming a therapeutic objective of exogenous remyelination, the precise stage of *ex vivo* lineage commitment is of critical importance. This is illustrated by the demonstration in animal models (attempting to achieve exogenous remyelination) that mature oligodendrocytes are ineffective whereas oligodendrocyte progenitor cells (OPCs), (e.g., hESC-derived) succeed and remyelinate [93]. These issues highlight several important concepts in cellular repair strategies that may affect the therapeutic potential of the cell type in question, including through altering post-transplantation cell fate. Further understanding of the interplay between pre-implantation differentiation state (*i.e.*, the degree of lineage commitment) and the effects of host environment on post-implantation lineage subspecification are thus critical if the promise of stem cells is to be realised in the clinical arena.

### 6.2. Promotion of Endogenous Repair Mechanisms

Of these two strategies, promoting endogenous repair may prove more clinically realistic and practically feasible in the near future. Specifically, given that repopulation of MS plaques with functional OPCs is robust, the virtue of transplanting OPCs into such lesions is questionable and may be unnecessary. As endogenous remyelination can be extensive [14,15] and resident OPCs robustly repopulate demyelinated lesions, an ideal approach to enhancing remyelination is to target endogenous repair. A pre-requisite to this approach, however, is elucidating the molecular mechanisms of remyelination and its ultimate failure in order to identify therapeutic targets.

In MS lesions, rather than addition of more OPCs, it is the inhibitory lesion environment that needs to be manipulated into one that is more permissive for remyelination. *Ex vivo* pre-transplantation manipulation of stem cells is a possible strategy here; but it seems more intuitive to engage the abundant endogenous OPC population (possibly through exogenous stem cells). Against that background, a further noteworthy generic attribute of stem cells is the capacity to dynamically interact with the microenvironment they migrate to in a manner that may be beneficial to the host, or “therapeutic plasticity”. Promoting endogenous repair might also be achieved by transplanting engineered stem cells that secrete growth factors (e.g., platelet derived growth factor [155]) or targeted drug delivery (e.g., fibroblast growth factor pathway antagonist [156]) that exploits the innate attraction of stem cells to areas of pathology (a generic property called “pathotropism”).

Stem cells can also be isolated from non-neural tissues throughout life (e.g., mesenchymal stem cells isolated from bone marrow). Although these cell populations do not reliably generate functional neural derivatives, their therapeutic potential arises from their biological properties through either the direct constitutive actions of the cells in question, such as immunomodulatory, or through their use as a vehicle following *ex-vivo* manipulation to secrete growth factors as mentioned above. A recent clinical trial has confirmed that autologous MSCs can be safely administered intravenously in secondary progressive MS with some evidence to suggest structural, functional and physiological improvement following treatment consistent with neuroprotection [157]. It is likely that any improvement in this context was achieved through different indirect mechanisms (e.g., immunomodulation), harnessing endogenous repair. Clearly such studies need to be repeated with larger numbers of patients, but do already provide reason for cautious optimism.

## 7. Concluding Remarks

Current therapeutic strategies for MS focus on symptomatic treatment and have only limited scope for arresting disease progression, let alone restoring structure and function where neurological deficit has accumulated. There is a large and unmet clinical need for new treatments in MS, particularly in the progressive phase. A key objective in terms of understanding the underlying molecular pathology, is to unravel the interrelationship of inflammation and axonal loss in MS; the key therapeutic objective to prevent axonal loss and limit demyelination. An intuitive strategy is to promote remyelination to achieve axonal protection. Recent advances in human stem cell biology have given rise to unprecedented experimental opportunities to study neurodegenerative disease using clinically relevant model systems, and patient-derived iPSCs now offer an unparalleled human system for *in vitro* modeling of disease mechanisms and discovering new therapeutic strategies. Proof-of-concept studies have already demonstrated the practical feasibility of using both mouse and human *in vitro* iPSC model systems to elucidate both cell-autonomous and non-cell-autonomous mechanisms of neurodegeneration [131–133]. At present, the main utility of human stem cell approaches therapeutically would appear to be indirect, through the promotion of endogenous repair mechanisms, rather than direct cellular replacement. All existing data converge on the promotion remyelination as being the most effective principal therapeutic strategy to slow down, stop and reverse functional deficits arising in MS. Human stem cells possess all desirable attributes to help realize this promise, both in terms of understanding disease/repair mechanisms but also through promotion of remyelination (via different direct and indirect mechanisms). Future research should focus on how to optimally harness these attributes to maximally exploit their therapeutic potential for the benefit of MS patients.

## Figures and Tables

**Table 1:** Some of the current animal models of MS.

Animal model	Phenotype	Main utility/comments
**Myelin mutant** e.g., Shiverer (myelin basic protein, MBP) Rumpshaker (proteolipid protein, PLP) Jimpy (proteolipid protein, PLP) Myelin associated glycoprotein (MAG) knockouts	Dysmyelination Altered neurotransmission In some cases “clinical” disease	To study myelination and/or related axonopathy
**Toxin based** e.g., Cuprizone Ethidium bromide Lysolecithin MAG-knockouts	Focal toxic demyelination	To study demyelination and remyelination
**Viral models** e.g., Semliki Forest Virus Theiler’s Murine Encephalomyelitis Virus Canine distemper Visna infection of sheep Infection of non-human primates	Viral/autoimmune demyelinating disease	To study demyelination and remyelination
**Autoimmune models** e.g., Experimental allergic encephalomyelitis (EAE); many subtypes, including active and adoptive-transfer in mice or rats.	Autoimmune demyelination Monophasic or relapsing-remitting.	This represents the main model system used. Major differences exist between EAE and MS; EAE requires active sensitization with brain antigens where as MS is a spontaneous disease. Spontaneous models of EAE have been developed but these necessitate transgenic approaches +/− strong immune adjuvants.
